# A case of dupilumab used to treat the exaggerated response to insect bites in the setting of chronic lymphocytic leukemia

**DOI:** 10.1016/j.jdcr.2024.01.003

**Published:** 2024-01-17

**Authors:** William Guo, Devin Miller, Rachel Manci, Adam Korzenko

**Affiliations:** Department of Dermatology, Stony Brook University Medical Center, Stony Brook, New York

**Keywords:** case report, chronic lymphocytic leukemia, CLL, dupilumab, dupixent, exaggerated response to insect bites, insect bites

## Introduction

Exaggerated response to insect bites is a rare cutaneous manifestation associated with chronic lymphocytic leukemia (CLL). It is characterized by a nonspecific cutaneous eruption after insect bites in patients with CLL. Lesions can present with, edema, erythema, pruritus, and even bullae.^1^^,^^2^ Treatment of the underlying CLL often does not adequately treat the exaggerated cutaneous reaction. Common treatments used for the cutaneous response include topical and intralesional steroids, oral steroids, rituximab infusions, dapsone, and intravenous immunoglobulins (IVIGs).^3^^,^^4^ We report the case of a patient with CLL exaggerated cutaneous response to insect bites treated with dupilumab.

## Case report

**Fig 1:**
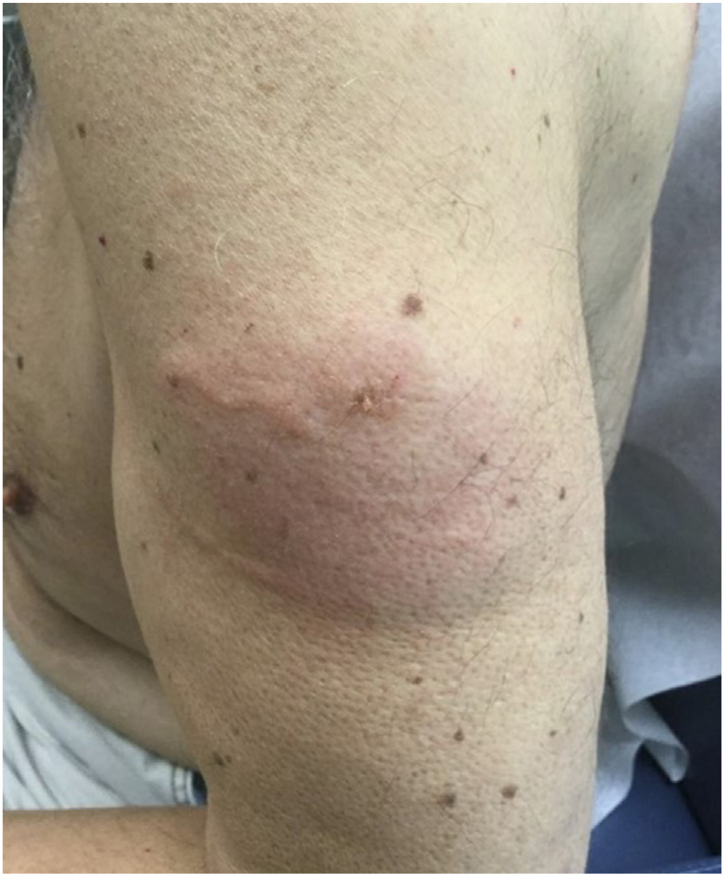
Edema and blister formation in a patient with chronic lymphocytic leukemia after insect bite.

**Fig 2:**
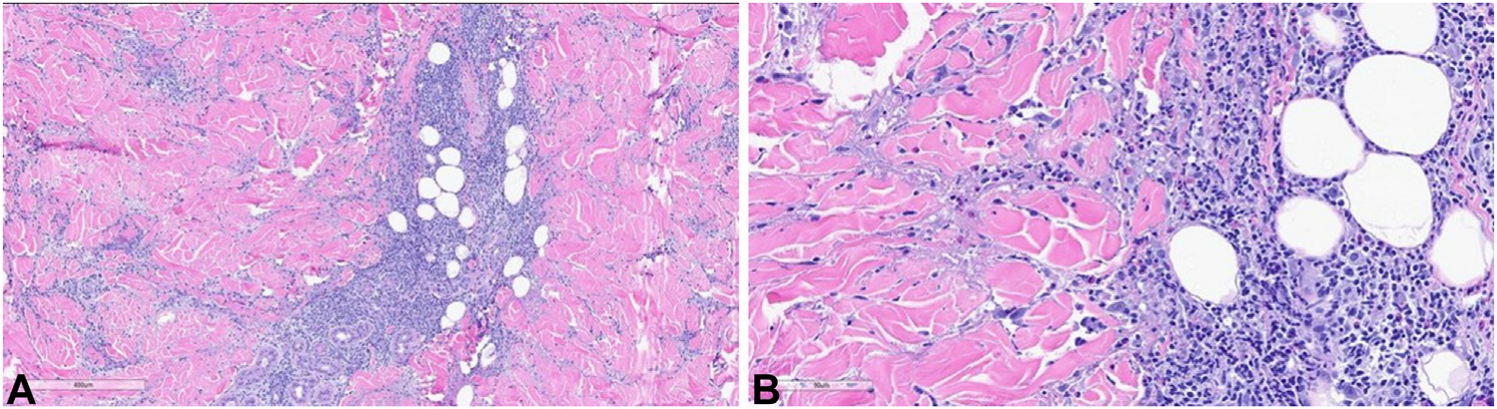
**A,** Histology showing papillary and reticular perivascular and interstitial dermatitis with numerous eosinophils, ulcerated. **B,** Higher power histology image showing papillary and reticular perivascular and interstitial dermatitis with numerous eosinophils, ulcerated.

A 55-year-old man presented to our dermatology clinic with a 2-year history of extreme cutaneous reactions to insect bites. The patient would experience marked edema and erythema that developed days after witnessed insect bites. This would be occasionally followed by blistering and resultant skin ulcerations that healed with scarring (Fig 1). The reaction to insect bites adversely affected the patient’s quality of life and bites were largely unavoidable because of the patient’s occupation as a landscaper. The patient’s medications included bupropion, omeprazole, budesonide-formoterol, and trazodone. The patient weighed 90 kg. The patient endorsed a recent diagnosis of CLL with the onset of the extreme cutaneous reaction to insect bites coinciding with CLL diagnosis. Other medical history included depression, gastroesophageal reflux disease, and asthma. Skin biopsy was performed that displayed an ulcer with papillary and reticular perivascular and interstitial dermatitis with numerous eosinophils (Fig 2). Direct immunofluorescence was completed to rule out bullous pemphigoid and was negative. Laboratory findings were significant for anemia with hemoglobin of 10.7 g/dL and absolute eosinophilia to 872 cells/μL. Diagnosis was made of exaggerated response to insect bites in the setting of CLL.

**Fig 3:**
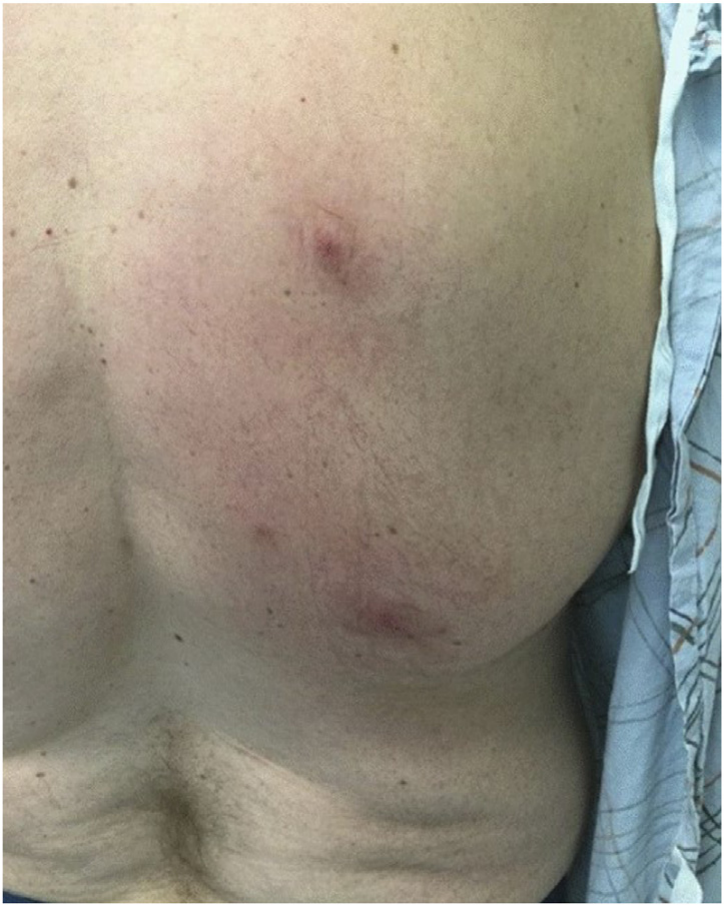
Decreased edema in new insect bites 1 month after starting dupilumab.

**Fig 4:**
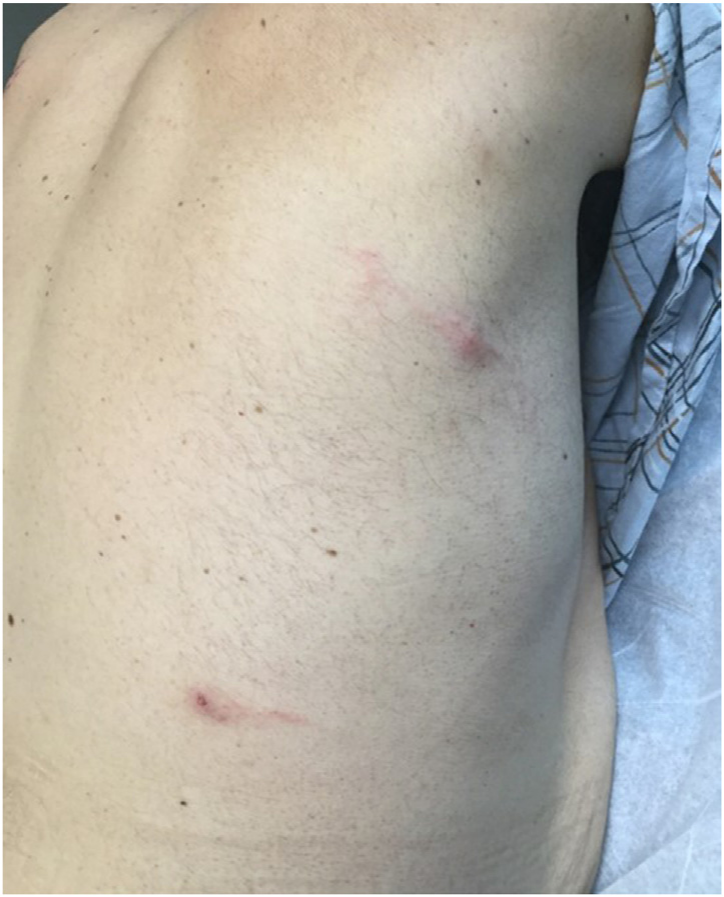
Insect bites 4 months after starting dupilumab.

The patient was started on a combination of oral antihistamines and betamethasone ointment for flares. This combination treatment did not improve existing lesions or prevent new lesions from forming. Prednisone tapers (60 mg for 5 days, then 40 mg for 5 days, then 20 mg for 5 days then stop) were given 2 times a few months apart during the first year, which were effective in resolving lesions; however, a more long-term treatment was necessary as patient had frequent flares that would have necessitated constant use of systemic corticosteroids. Oral dapsone titrated to 150 mg daily (starting with 100 mg daily for 1 month before increasing to 150 mg daily for 2 more months) did not achieve any response in the patient. At 2 years into treatment, the patient achieved stability in his CLL with hematology; however, his adverse reaction to insect bites continued. Patient was subsequently started on rituximab infusions with hematology, which also did not affect disease activity. IVIG infusions were also attempted with hematology that helped the patient’s anemia but did not affect his cutaneous disease. After failing known therapy, the decision was made in conjunction with hematology to trial the patient on dupilumab injections. Dupilumab was administered with a 600 mg loading dose followed by 300 mg injections every 2 weeks, following the dosing guidelines in adult atopic dermatitis. At his 1-month follow-up, the patient was still experiencing adverse reactions to insect bites; however, he believed that the reactions were diminished and his lesions were healing faster (Fig 3). He was not using topical or oral steroid therapy at the time. At his 4 month follow-up, the patient was happy to report that he had not had an adverse reaction to an insect bite in over 1 month, and prior areas of reaction had healed (Fig 4). Some areas of ulceration healed with scarring. His anemia stayed stable. No side effects from dupilumab were noted. Patient was continued on dupilumab therapy and the dose was decreased to 300 mg every month without recurrence of cutaneous reactions.

## Discussion

To our knowledge, this is the first reported case of exaggerated cutaneous response to known insect bites in CLL treated successfully with dupilumab. Exaggerated response to insect bites and insect bite-like reactions in the absence of actual arthropod bites are 2 rare cutaneous manifestations in patients with hematologic malignancies. One prior case described the successful treatment of insect bite-like reactions with dupilumab.^5^ CLL is the most commonly associated hematologic malignancies with these conditions.^5^^,^^6^ The presentation of skin lesions is variable, ranging from edema and erythema to hemorrhagic bullae.^2^ The exaggerated response to insect bites does not always correlate with disease activity of CLL, and in some cases will actually precede diagnosis of CLL. Our patient had quiescent CLL but still had an exaggerated response to insect bites.

Treatment of exaggerated response to insect bites has traditionally included topical and intralesional steroids, oral steroids, rituximab infusions, dapsone, and IVIG.^3^^,^^4^ Oral steroids have been reported to have the best efficacy but the disease will often recur after the dose is tapered, which matches what happened in our patient. Our patient failed all therapies except for oral corticosteroids, and because of the long-term side effects of systemic steroids, alternative therapy was desired. Dupilumab administered at the standard dose for adult atopic dermatitis proved effective. The patiuent reported no side effects from the drug. Results were seen as early as 1 month after beginning therapy. In addition, clinical resolution was maintained when decreasing the frequency of dosing to 300 mg every month.

Weed^1^ hypothesized that the pathogenesis of the exaggerated response to insect bites in CLL was because of neoplastic B cells causing increased interleukin (IL)-4 and IL-5 production. Dupilumab is a monoclonal antibody treatment known to relieve the cutaneous manifestations of atopic dermatitis, which acts by binding to the alfa subunit receptor of IL-4, thereby inhibiting the signaling pathways of IL-4 as well as IL-13 that plays a central role in the regulation of immune responses.^2^ By blocking IL-4 and IL-13, dupilumab is likely able to reduce the inflammatory process and decrease the recruitment of eosinophils to the skin lesion.

In conclusion, dupilumab should be considered for the treatment of recalcitrant exaggerated response to insect bites in patients with CLL. Treatment may be effective even when decreasing dosage to 300 mg monthly.

## Conflicts of interest

None disclosed.
